# Dendrimeric peptides can confer protection against foot-and-mouth disease virus in cattle

**DOI:** 10.1371/journal.pone.0185184

**Published:** 2017-09-26

**Authors:** Ivana Soria, Valeria Quattrocchi, Cecilia Langellotti, Mariela Gammella, Sebastian Digiacomo, Beatriz Garcia de la Torre, David Andreu, Maria Montoya, Francisco Sobrino, Esther Blanco, Patricia Zamorano

**Affiliations:** 1 Instituto de Virología, Centro de Investigaciones en Ciencias Veterinarias, Instituto Nacional de Tecnología Agropecuaria (INTA)-Castelar, Buenos Aires, Argentina; 2 Consejo Nacional de Investigaciones Científicas y Técnicas (CONICET), Buenos Aires, Argentina; 3 Departament de Ciencies Experimentals i de la Salut, Universitat Pompeu-Fabra, Barcelona, Spain; 4 The Pirbright Institute, Ash Road, Woking, Surrey, United Kingdom; 5 Centro de Biología Molecular “Severo Ochoa” (CSIC-UAM), Madrid, Spain; 6 Centro de Investigación en Sanidad Animal (CISA-INIA), Valdeolmos, Madrid, Spain; Instituto Butantan, BRAZIL

## Abstract

Foot-and-mouth disease virus (FMDV) causes a highly contagious disease in cloven-hoofed animals. A synthetic vaccine candidate consisting of dendrimeric peptides harbouring two copies of a B-epitope [VP1(136–154)] linked to a T-cell epitope [3A(21–35)] of FMDV confers protection to type O FMDV challenge in pigs. Herein we show in cattle that novel dendrimeric peptides bearing a T-cell epitope [VP1(21–40] and two or four copies of a B-cell epitope [VP1(135–160)] from type O1 Campos FMDV (termed B_2_T and B_4_T, respectively) elicited FMDV specific immune responses to similar levels to a commercial vaccine. Animals were challenged with FMDV and 100% of vaccinated cattle with B_2_T or B_4_T were protected to podal generalization. Moreover, bovines immunized with B_4_T were completely protected (with no clinical signs) against FMDV challenge after three vaccine doses, which was associated with titers of viral neutralizing antibodies in serum higher than those of B_2_T group (p< 0.05) and levels of opsonic antibodies similar to those of animals immunized with one dose of FMDV commercial vaccine. Bovines vaccinated with both dendrimeric peptides presented high levels of IgG1 anti FMDV in sera and in mucosa. When IgA in nasal secretions was measured, 20% or 40% of the animals in B_2_T or B_4_T groups respectively, showed anti-FMDV IgA titers. In addition, B_2_T and B_4_T peptides evoked similar consistent T cell responses, being recognized *in vitro* by lymphocytes from most of the immunized cattle in the proliferation assay, and from all animals in the IFN-γ production assay. Taken together, these results support the potential of dendrimers B_2_T or B_4_T in cattle as a highly valuable, cost-effective FMDV candidate vaccine with DIVA potential.

## Introduction

Foot-and-Mouth disease virus (FMDV) is a Picornavirus belonging to the genus Aphthovirus. The viral particle consists of a positive-strand RNA genome encoding four capsid proteins, VP1, VP2, VP3 and VP4, and eleven different mature non-structural proteins, NSP-3A among them [1]. FMDV has seven immunologically distinct serotypes, namely O, A, C, SAT1, SAT2, SAT3 and Asia 1. This virus produces a highly transmissible and devastating disease of farm animals, foot-and-mouth Disease (FMD), which is the most feared animal disease worldwide, causing large economic losses during an outbreak.

There is great disparity in progress towards FMD control and eradication. While some countries are either FMD free or well on the road to achieving eradication, others are at an early stage of FMD control. In endemic areas, such as Asia Africa and South America, FMD control is performed by regular vaccination based on inactivated whole-virus. [1]. The limitations shown by these conventional vaccines have prompted the study of new, safer alternative vaccines [2]. Synthetic peptides are one of the most promising vaccine candidates for infectious disease, such as FMD, as they are highly pure, defined, stable and safe. Moreover, recent studies have shown that synthetic peptides achieve protective immunity against challenge in swine [3, 4]. On the other hand, differentiation between vaccinated from infected animals is feasible with such vaccines.

Linear peptides containing an immunodominant B-cell site located in the GH loop around at positions 140–160 of capsid protein VP1 [5, 6] can protect against FMDV challenge. However, the protection conferred by these peptides is limited due, among other factors, to the lack of T-cell epitopes [5, 7]. Therefore, an effective peptide vaccine needs multivalency of B-cell epitopes to elicit a high neutralizing antibody response and T-cell epitopes that provide adequate cooperation [8]. Multiple antigenic peptides (MAPs), also named dendrimers, are radial or wedge-like branched macromolecules that contain a peptide core attached to a define number of epitopes [9, 10]. Pigs vaccinated with a dendrimeric peptide, which included the T-cell epitope 21–35 from 3A and four copies of the B-cell site VP1 [136–154], were protected against type C FMDV challenge [3]. Recent studies indicated that a similar construct incorporating the B cell site sequence from type O FMDV O-UKG induced full protection in pigs [4].

In this report, novel dendrimeric peptides were designed containing a bovine T-cell epitope 21–40 from VP1 [11] linked to two (B_2_T) or four (B_4_T) copies of the B-cell site VP1 [135–160] from FMDV O1 Campos assembled using a maleimide conjugation. A pilot study was performed to address the immunogenicity of such dendrimeric vaccines and the protection afforded in cattle against viral challenge.

Our results show for the first time that not only tetravalent presentation of B-cell epitopes linked to the T-bovine epitope but also bivalent formulation results in an effective vaccine that conferred protection in cattle. Thus, these synthetic peptides can be considered promising vaccine candidates with reasonable prospects of clinical application.

## Materials and methods

### Dendrimeric peptides

**Fig 1:**
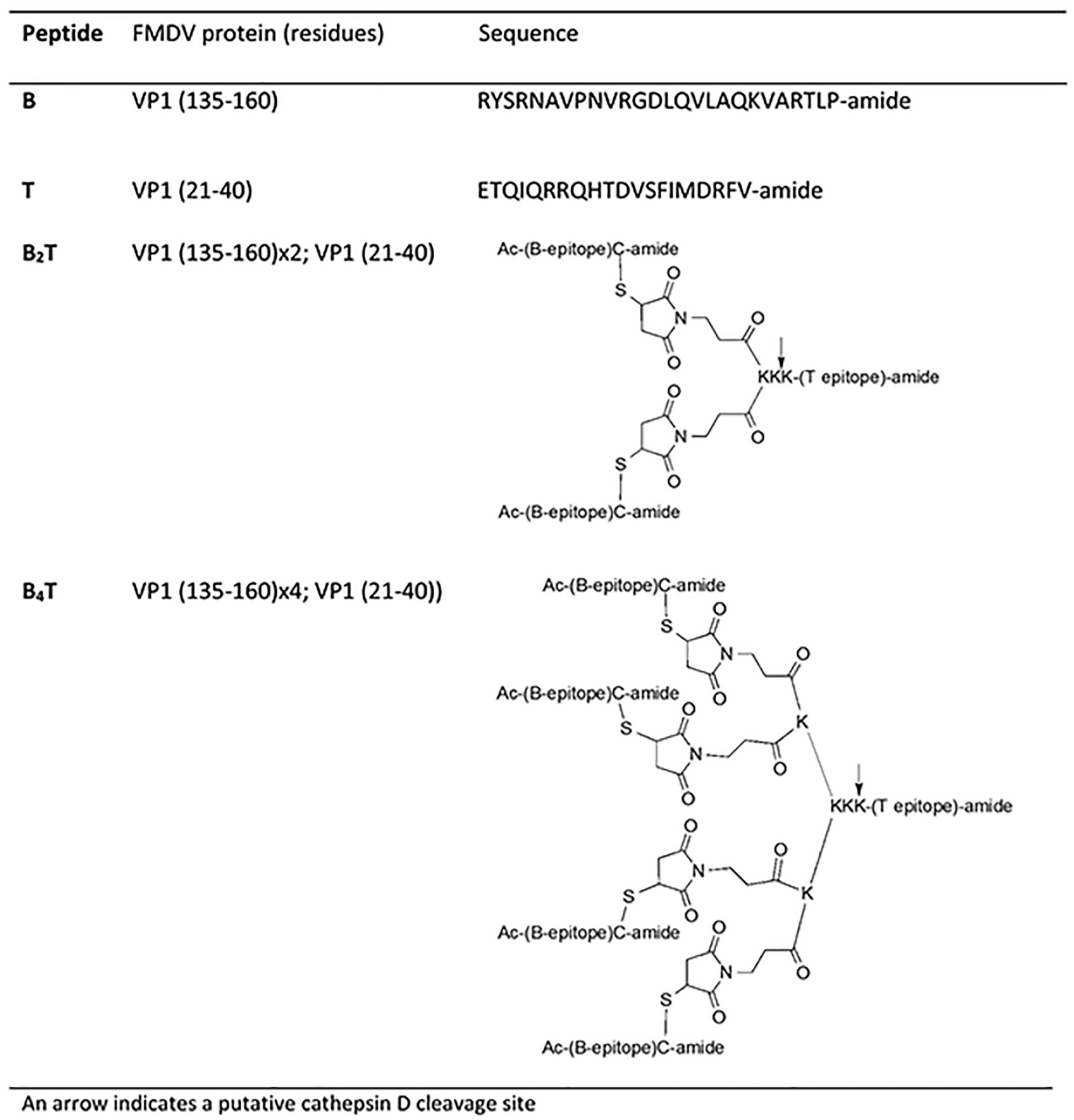
Dendrimeric peptides used in this study.

Two peptides (Fig 1) incorporating a T-cell epitope [VP1 (21–40)] immunodominant for bovine lymphocytes [11, 12] and two or four copies of the B-cell epitope [site A–VP1 (135–160)], termed B_2_T or B_4_T, respectively, in a dendrimeric arrangement were synthesized as described [4, 13]. The C-terminal Cys side chain thiol is linked to Lys via a 3-maleimidopropionic acid unit; the peptides were purified by HPLC and characterized by mass spectrometry. Sequences are derived from FMDV O1 Campos.

### Virus

FMDV O1 Campos was kindly provided by Biogenesis Bagó S.A. as binary ethylene-imine (BEI)-inactivated (iFMDV). Purified virus was obtained by a sucrose density gradient centrifugation method [14] and was used for ELISA and lymphoproliferation assay. For challenging and virus neutralization assays, infective virus (kindly donated by the Argentine National Service of Animal Health) was used in BSL-4 OIE laboratories and boxes at INTA.

### Animals, vaccines, immunization and infection of cattle

Eighteen Hereford calves serologically negative for FMDV, approximately 6 months old, were used in the experiment. Groups of five animals were inoculated twice (day 0 and 18) by subcutaneous injection in the front left quarter with 2 mg of B_2_T or B_4_T peptide in 2 ml of a water-in-oil single emulsion. The adjuvant included was MONTANIDE ISA 50 V (Seppic). At 38 dpv animals were boosted with 0.5 mg of B_2_T or B_4_T peptide. Forty-six days after the first vaccination, immunized animals as well as 3 control unvaccinated bovines were challenged by nasal instillation with 1 ml (0.5 ml for each nostril) of a 10^4^ suckling mouse lethal doses 50% equivalent to 10^4^ Bovine Infective Doses 50% (BID_50_) of infective FMDV O1 Campos (determined by titration on cattle tongue) [15–17]. This method mimics a natural FMDV infection [18]. Control unvaccinated bovine (n = 3) were challenged at the same time and following the same procedure. Seven days post challenge (dpc) all animals were checked for FMDV-induced lesions on feet, mouth and tongue. Animals with absence of FMDV lesions at the feet were considered protected against podal generalization (PPG). Another five calves were immunized by subcutaneous injection with a single dose of commercial FMDV vaccine, a water in oil single emulsion containing FMDV strains A Arg 2000, A Arg 2001; A24 Cruzeiro and O1 Campos. This vaccine had been approved by the Argentine Animal Health Service (SENASA) with more than 80% of expected percentage of protection against each of the viruses included in its formulation [19]. This group was used as a control of immune response quality. Experiments were carried out according to INTA Ethics Manual “Guide for the use and care of experimental animals”. The protocol was approved by the Institutional Animal care and use Committee “Comité Institucional para el cuidado y uso de animales de experimentación” CICUAE INTA CICVyA (Permit Number: 77/2013).

### Measurement of anti FMDV antibodies

An indirect ELISA was used for anti-dendrimer antibodies measurement. Maxisorp 96-well plates (Nunc) were coated with B_4_T peptide (30μg/ml), plates were washed and blocked with PBST-OVA 1% and dilutions of serum samples were added. After incubation, plates were washed and horseradish peroxidase (HRP) labeled goat anti-bovine IgGs antibody (KPL, USA) was added. After washing, ortho-phenylenediamine (OPD)-H2O2 was added as HRP substrate. FMDV-specific antibodies were detected by means of an indirect ELISA, as described [20]. Briefly, Immulon II 96-well ELISA plates were coated with 2.6 μg/ml FMDV O1Campos and processed as described above.

The antiviral ELISA detailed above was modified in order to detect FMDV-specific IgG1, IgG2 (in sera), and IgG1, IgA (in nasal swabs) antibodies [20, 21]. After incubation with samples, a mouse anti-bovineIgG1, IgG2 or IgA monoclonal antibody was added (kindly provided by Dr. S. Srikumaran, University of Nebraska, USA). Lastly, a (HRP) labeled goat anti-mouse IgG antibody was added after wash. OPD was used as HRP substrate. Absorbance was recorded at 492nm (A492) in a microplate photometer (Multiskan FC, Thermo). The cut-off was established as the mean A492 of the negative sera (from 5 unvaccinated animals) plus two standard deviations (SD). Antibody titers were calculated as log _10_ of last reciprocal dilution above cut-off. Positive control sera were included in every plate.

### Opsonophagocytosis assay

Inactivated FMDV (iFMDV) was labeled with FITC (Sigma, St. Louis,MO) as described [22]. Briefly, FITC-iFMDV was incubated with serum and added to bovine macrophage cells (BoMac) [23]. Prior to inactivation, the virus was titrated, by Reed and Müench method, in order to incubate the BoMac cells with a quantity of virus representing a multiplicity of infection of 10. Extracellular fluorescence was quenched with Trypan Blue. The ability of the antibodies to opsonize viral particles was analyzed by flow cytometry, using a FACSCalibur (Becton Dickinson, San Jose, CA) and CellQuest software. Results were expressed as % of BoMac cells incorporating FITC-iFMDV after incubation with sera. The cut-off was calculated as the mean percentage of BoMac cells incorporating FITC-iFMDV incubated with five negative sera, plus 2 SD.

### Percentage of FMDV neutralization

The percentage of virus neutralized by serum from immunized cattle, at 38 and 44dpv (upon 2 and 3 peptide doses, respectively) was measured as previously describe [24] with minor modifications. Briefly, a 1/8 serum dilution was incubated with different 10-fold dilutions of infective FMDV (1000 to 1 TCID_50_), and the infective virus recovered was determined by a TCID_50_ assay. Titers were expressed as the % of the initial virus neutralized upon incubation. Significant differences are indicate as *** (p<0.001).

### Serum Neutralization titer

Serum samples were examined for anti-FMDV neutralizing antibodies (fixed virus, variable serum) as described before [20]. Briefly, serial dilutions of inactivated sera were incubated for 1 h at 37°C with 100 TCID_50_ of infective FMDV. Then virus-serum mixtures were seeded on BHK-21 monolayers. After 40 min at 37°C, fresh medium supplemented with 2% FCS was added to the cells that were incubated at 37°C under 5% CO_2_. Cytopathic effects were observed after 48 h. Titers of virus neutralizing antibodies were expressed as log_10_ of the reciprocal of the serum dilution which neutralize 50% of 100 FMDV DICT_50_.

### Lymphoproliferation assay

Peripheral blood mononuclear cells (PBMC) were obtained from cattle as described [25] and 2x10^7^ cell/ml were labeled with carboxyfluorescein diacetate succinimidyl ester (CFSE) 3 μM in PBS for 15 min at 37°C. Labeled cells were added to 96-well plate (5 x 10^5^ cell/well) containing (i) 5μg/ml iFMDV, (ii) 50μg/ml of peptides B_2_T, B_4_T or T, and (iii) 5μg/ml Concanavalin A (Sigma–Aldrich, S t. Louis, MO). Cells were incubated at 37°C in 5% CO_2_ atmosphere 5 days, then 0.2% paraformaldehyde was added and cell proliferation was analyzed by flow cytometry. Results were expressed as %CFSE proliferation. The cut-off was the mean % CFSE proliferation in wells without stimuli plus 2 SD.

### IFN-γ detection

PBMC were cultured with 50μg/ml of B_2_T, B_4_T or T peptides for 72 h. Supernatants were analyzed using ELISA as described previously [20]. Briefly, plates were coated with a mAb against- IFN-γ (kindly donated by Dr. L. Babiuk). Samples and recombinant IFN-γ standard (Serotec, UK) were added and IFN was detected using rabbit polyclonal anti-IFN-γ antibodies. After incubation, biotinylated goat anti-rabbit IgG antibody was added and then, HRP-conjugated streptavidin (KPL, USA). Plates were washed, incubated with (OPD)-H2O2 and read at 492nm. IFN-γ concentration was calculated from interpolation of data in the standard curve.

### RNA extraction and PCR amplification of viral RNA

Samples were obtained from lesions of the disease on tongue and viral RNA was extracted using Trizol (Invitrogen) and used as template of a reverse transcription reaction performed with random primers. The resulting cDNA was used to carry out PCR amplification of, on the one hand, the FMDV 3D polymerase gene (primers: GK2 -antisense- CTAGACCGTGTTGGTGGGTT and GK7 -sense- CCGACCACCACGGTGTTTTCG) and of host 18S ribosomal RNA as internal control. Amplicons of 380 bp and 480 bp were obtained for 3D viral polymerase and host rRNA, respectively. On the order hand, PCR amplification of FMDV VP1 gene (primers: P12B -antisense- TTCGAAGTACCAGGGTTTGGC and ALFA1 -sense- CTCGTTCATCATGGACAGATT). Amplicons of 780pb were obtained and direct sequencing of the PCR products was performed by capillary electophoresis.

### 2.11 Statistical analysis

One-way analysis of variance (ANOVA) and Bonferroni post-tests were used to compare data between three or more groups.

## Results

### B_2_T and B_4_T design and synthesis

B_2_T and B_4_T were designed as novel vaccine candidates as described in Fig 1. These new formulations displayed either two or four copies of the B-cell epitopes VP1 [135–160] [26] together with a single copy of the bovine T-cell epitope VP1 [21–40] [11, 12] both derived from FMDV O1 Campos and were produced as described in 2.1.

### B_2_T and B_4_T induce anti-peptide and FMDV specific antibodies

**Fig 2:**
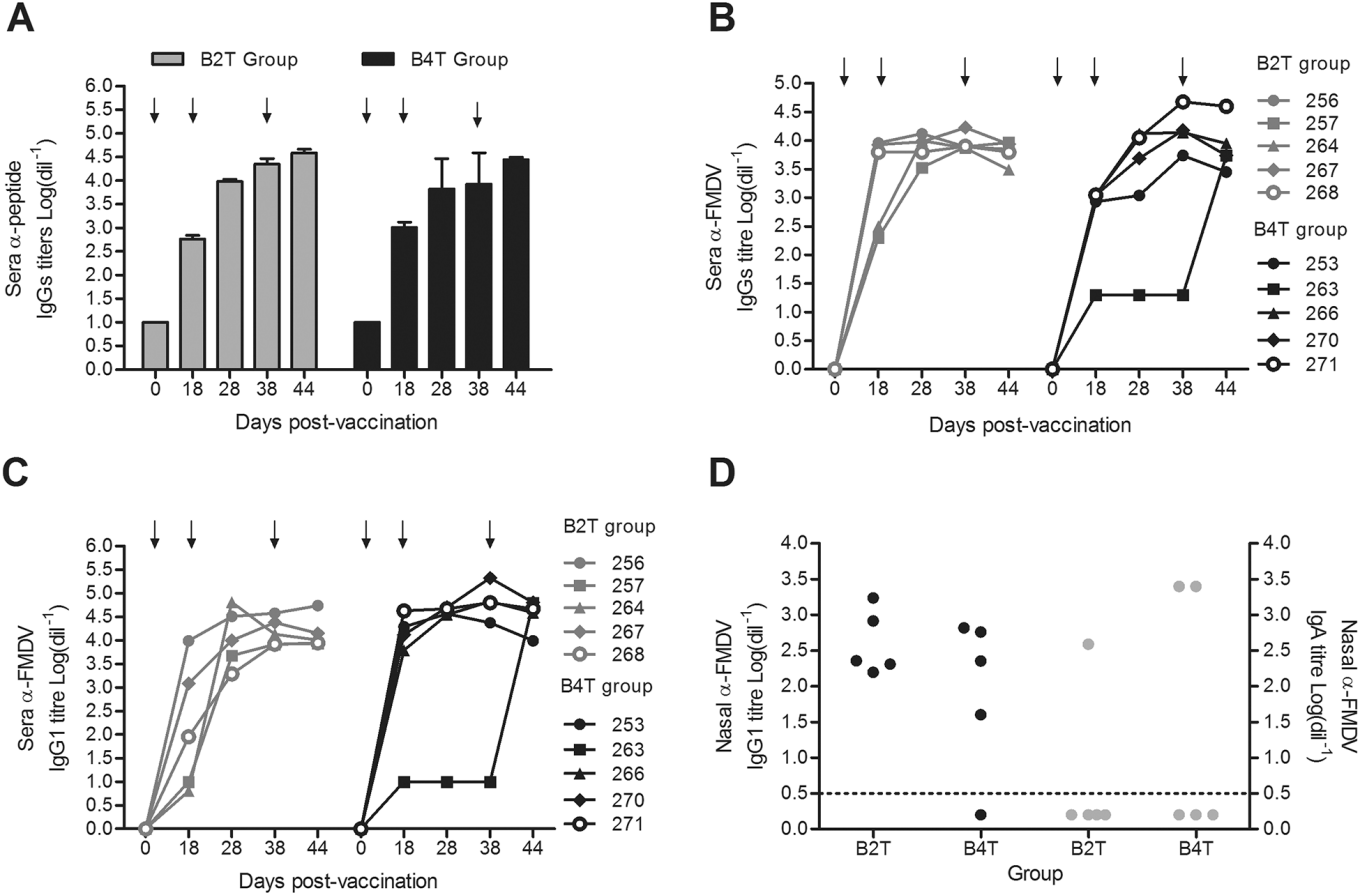
Antibody detection by ELISA in vaccinated cattle. Animals were immunized on day 0, 18 and 38 (arrow) with B_2_T or B_4_T vaccine. (A) Kinetics of anti-peptide serum antibodies. (B, C) Kinetics of total IgG anti-FMDV and IgG1 anti-FMDV serum antibodies. Titers were calculated as log _10_ of last reciprocal dilution above cut-off. (D) FMDV-specific mucosal IgG1 and IgA response. Nasal swabs were collected at 28dpv. Each point represents the nasal IgG1 or IgA anti-FMDV antibody titers (log_10_) of each animal. Cut-off was established as the mean value of mock vaccinated animals plus twice the standard deviation value.

All animals inoculated with either B_2_T or B_4_T constructs developed specific and pronounced anti-peptide as well as anti-FMDV total IgG and IgG1 responses (Fig 2A, 2B and 2C), including animal 263 that showed increased levels of antibodies against FMDV after the third peptide dose (Fig 2B). Bovines 257 and 264 (B_2_T) presented high levels of IgG1 only after the second peptide dose (Fig 2C). At 44dpv, high anti-FMDV IgGs titers were detected in all animals (3.9±0.1 and 4.0±0.2 in B_2_T and B_4_T groups, respectively) (Fig 2B). Low levels of specific FMDV IgG2 were detected in both B_2_T and B_4_T groups with average antibody titers of 1.36±0.25 and 1.5±0.4, respectively (data not shown).

### FMDV-specific mucosal immunity

Animals from B_2_T and B_4_T groups exhibited also high levels of anti-FMDV IgG1 in nasal secretions at 28dpv, with exception of bovine 263 that only showed high IgG1 titers (7±1.0) after the third immunization. When IgA was measured in nasal secretions, 1 out of 5 animals in the B_2_T and 2 out of 5 in the B_4_T group, showed anti-FMDV IgA titers (Fig 2D) indicating that these novel peptide constructs were able to induce not only systemic but also local mucosal immunity.

### Analysis of the opsonic capacity of the sera

**Fig 3:**
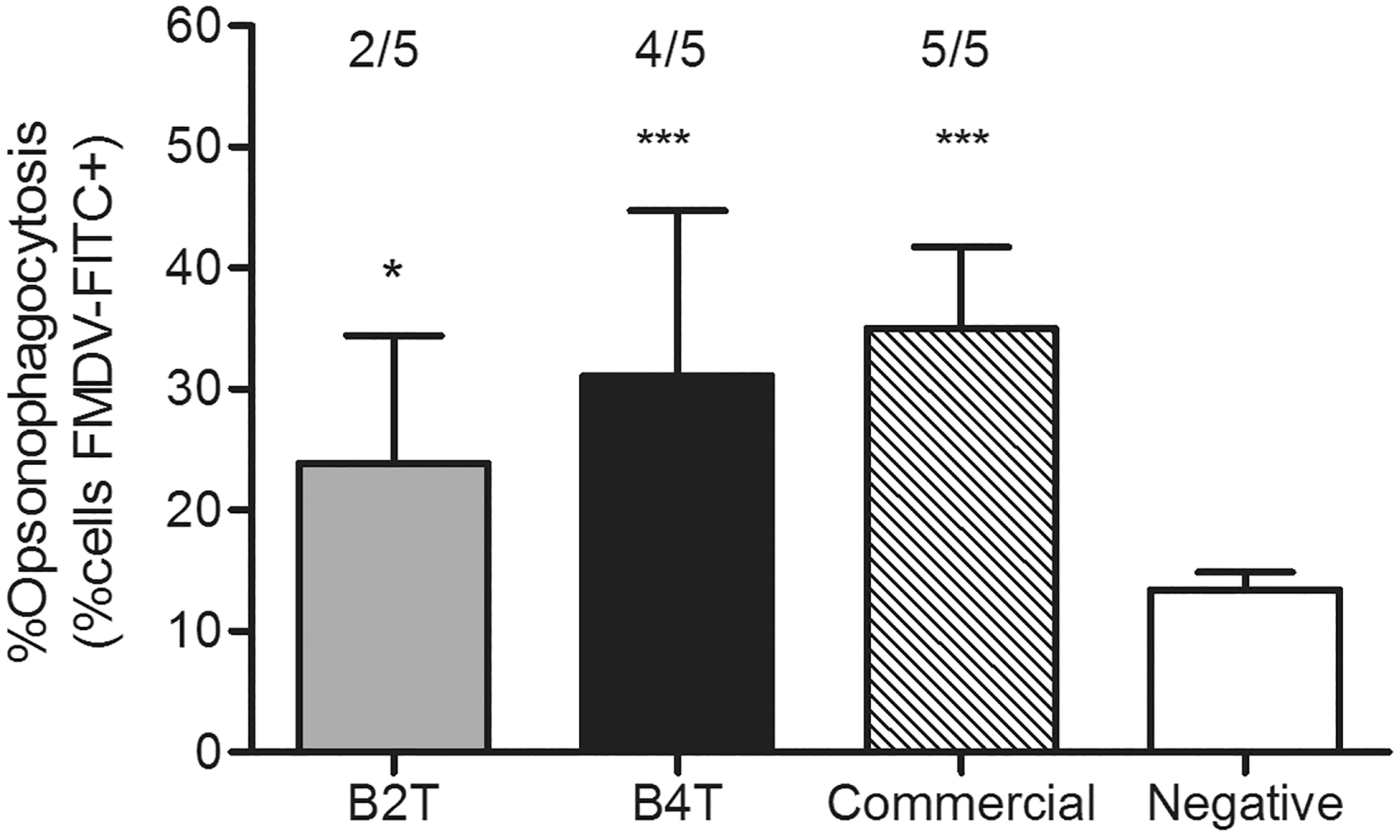
Opsonising capacity of sera (from 44 dpv) measured by flow cytometry. Results are expressed as the % of BoMac cells incorporating FITC-virus after incubation with sera from B_2_T, B_4_T, commercial vaccine or negative sera. Each bar represents the mean of opsonising capacity of sera + SEM. * and *** represents significant differences (p<0.05 or p<0.001, respectivelly) compared to opsonising capacity of negative sera (from day 0). The cut-off was calculated as the mean percentage of cells incubated with FITC-FMDV- and sera free of FMDV antibodies plus 2 SD. The number of positive sera/ the total number of sera analyzed are indicated above bars.

The importance of antibodies in FMD protection might be related to their ability to opsonize rather than to neutralize viral particles [27, 28]. The average values of opsono-phagocytosis of sera collected at the day of challenge (44dpv) were 24±11% in B_2_T group, 31±14% in B_4_T group and 35±6% in cattle immunized with an FMDV commercial vaccine (Fig 3). These values were significantly higher than those of sera before immunization (negative controls; 15±2%). In the B_2_T group, sera from 2 of the 5 animals showed significant responses, while 4 of the 5 animals in the B_4_T group responded positively. In general, a trend towards higher opsonization values was observed in sera from animals immunized with the B_4_T construct, similarly as the ones induced with the commercial vaccine.

### Analysis of the neutralizing capacity of the sera

**Fig 4:**
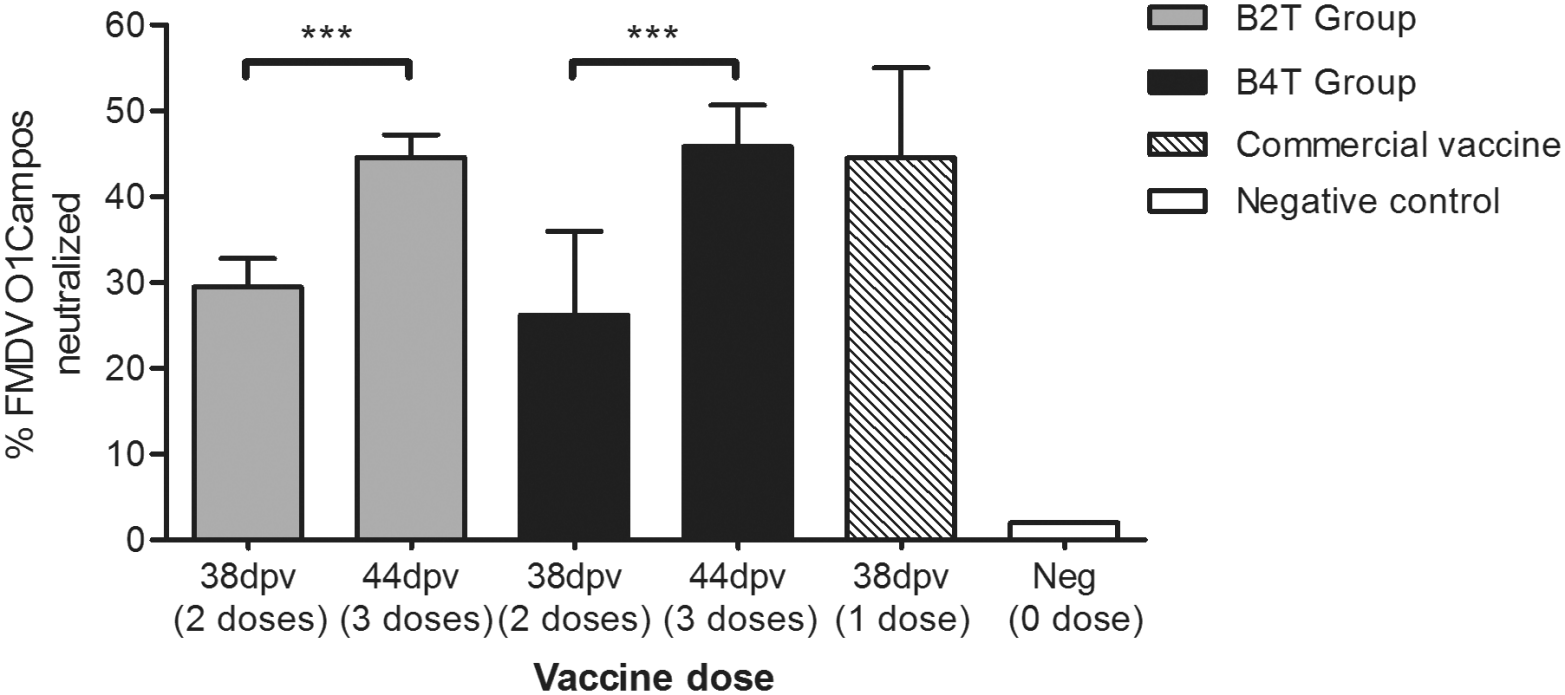
Neutralization of FMDV O1Campos by sera from vaccinated animals. (A) % of virus neutralized by serum from cattle immunized with B_2_T, B_4_T or a single dose of conventional vaccine, at 38 and 44dpv (upon 2 and 3 doses of peptide, respectively). A 1/8 serum dilution was incubated with different doses of FMDV O1 Campos, and the infective virus recovered was determined by a TCID50 assay. Titers are expressed as the % of the initial virus neutralized upon incubation. Significant differences from dose are indicate as ***(p<0.001).

We first determined the percentage of virus neutralized by a 1/8 dilution of sera from immunized cattle. At 38dpv, the average values were 29±3% in animals from B_2_T group and 26±10% in those from B_4_T group (Fig 4). However, when a virus neutralization test (VNT) was performed to detect the antibody dilution capable of neutralizing a fixed amount of virus, no serum sample at 38dpv was able to neutralize 100 TCID_50_ of FMDV O1Campos (VNT<0.9) (data not shown), supporting a weak induction of neutralizing antibodies in these animals. Taking into account these results, a third dose of each construct was administered to the calves that resulted in a significant increase at 44 dpv in the neutralizing capacity of sera (p<0.001) (Fig 4), being the percentages of neutralization 44±3% and 46±3% for animals in B_2_T and B_4_T groups, respectively. Remarkably, these values were similar to those of a bovine immunized with a single dose of commercial vaccine (45±10%). Interestingly, all animals were also positive for VNT, being the mean titers of antibodies in animals from B_4_T group (1.7±0.3) significantly higher (p<0.05) than those from B_2_T group (1.4±0.1). Again, these VNT values were similar to those observed in animals vaccinated with commercial vaccine (1.9±0.6) (Table 1).

**Table 1:** Virus neutralizing titers pre-challenge of cattle.

Group	Animal n°	VNT (44dpv)	Mean VNT
**B**_**2**_**T**	256	1.35	1.4 ± 0.1
	257	1.40	
	264	1.30	
	267	1.40	
	268	1.43	
**B**_**4**_**T**	253	1.20	1.7 ± 0.3
	263	1.65	
	266	1.80	
	270	2.00	
	271	1.97	
**Commercial vaccine**	692	1.8	1.9 ± 0.6
	700	2.1	
	709	1.6	
	710	2.3	
	722	2.8	
**Negative control**	254	<0.9	<0.9
	241	<0.9	
	261	<0.9	

Titer of virus-neutralizing antibody at day 44 post vaccination. Titers were expressed as log_10_ of the reciprocal of the serum dilution which neutralize 50% of 100 DICT _50_ of FMDV

### Specific cellular immune response and IFN-γ release in vaccinated animals

Before challenge, at 44dpv, specific *in vitro* lymphoproliferations were conducted using several stimuli. Significant values of proliferation (% CFSE proliferation ≥4) to the peptide used for immunization were found in 4 out of 5 animals of B_2_T group and in all the five animals from B_4_T group (Table 2A). Responses to dendrimers (B_2_T or B_4_T) not used for immunization were similar to those achieved with the immunizing peptide while the number of animals that recognized the T cell peptide alone was lower. In group B_2_T, PBMC from bovines 256 and 257 significantly proliferated in response to the T peptide and animal 267 showed no response to any stimulus. In group B_4_T only cells from bovine 263 and 271 proliferated when were stimulated with the T peptide. PBMC from a negative control animal (Table 2A) and from all bovines at day 0 did not respond to any peptide (data not shown).

**Table 2:** Cellular immune response of cattle at 44 dpv analyzed by lymphoproliferation and IFN-γ production.

Group	Animal n°	(A) % CFSE proliferation			(B) IFN-γ (x10^2^ pg/ml)		
		Ag: B_2_T	B_4_T	T	Ag: B_2_T	B_4_T	T
**B**_**2**_**T**	256	13.0	8.0	5.0	226.7	151	90
	257	15.0	16.0	20.0	119.7	143.4	85
	264	4.2	5.1	2.5	37	20.4	0.5
	267	2.0	1.0	1.0	6.8	4.7	2.9
	268	6.1	ND	1.0	12.6	8.5	6.4
**B**_**4**_**T**	253	5.1	3.0	2.0	15.1	15.1	0.5
	263	12.0	13	13	113.5	25.3	0.5
	266	8.2	7.1	1.0	14.1	20.9	4.3
	270	8.1	4.0	1.0	22.4	9.7	9.7
	271	4.1	9.0	6.1	52.1	42.4	0.5
**Negative control**	254	2.0	2.0	1.0	0.5	5.3	1.7

(A) Specific proliferation of PBMC from vaccinated bovines measured by CFSE labeling, the results are expressed as % CFSE proliferation. PBMC were stimulated *in vitro* with medium alone, or with peptides B_2_T, B_4_T, and T. For each peptide, the cut-off was calculated as the mean number of proliferating cells at 0 dpv plus 2 SD. Positive %CFSE proliferations are underlined (≥4).

(B) IFN-γ production by PBMC after peptide stimulation as in (A). The supernatants were tested by ELISA and the results, expressed in pg/ml, were calculated by interpolation in a cytokine standard curve. For each peptide, the cut-off was calculated as the mean IFN-γ production of PBMCs from animals at day 0 plus 2 SD. Positive IFN-γ production above cut-off were underlined (≥11.0 x 10^2^ pg/ml).

Levels of IFN-γ secreted by PBMCs from immunized animals were also determined at 44dpv (Table 2B). Positive IFN-γ responses were found in 4 out of 5 animals of B_2_T group and in all animals of B_4_T group following Ag-B_2_T stimulation. Whereas, Ag-B_4_T induced IFN-γ secretion in PBMCs from 3 animals of B_2_T group and from 4 animals belonging to the B_4_T group. In group B_2_T, only cells from bovine 256 and 257 secreted IFN-γ when they were stimulated with the T peptide, whereas only PBMCs from bovine 263 from group B_4_T animals recognized this peptide for IFN-γ secretion.

### B_2_T and B_4_T protect cattle against viral challenge

Subsequently, all animals were challenged at 44dpv by nasal instillation with infective FMDV O1 Campos and protection was measured by monitoring clinical signs in animals after challenge. As shown in Table 3, the three negative control animals showed typical FMDV lesions while, remarkably, all B_2_T and B_4_T vaccinated bovines were protected against podal generalization. Only animals 257 and 264, belonging to B_2_T group showed a single vesicle in tongue at 7dpc even though they were PPG. These two lesions were positive for FMDV RNA detection by RT-PCR (Table 3).

**Table 3:** Clinical lesions and protection post challenge of vaccinated cattle.

Group	Animal n°	Lesions		Protection
		Foot	Tongue	
**B**_**2**_**T**	256	−	−	PPG
	257	−	+**	PPG
	264	−	+**	PPG
	267	−	−	PPG
	268	−	−	PPG
**B**_**4**_**T**	253	−	−	PPG
	263	−	−	PPG
	266	−	−	PPG
	270	−	−	PPG
	271	−	−	PPG
**Negative control**	254*	+	**+**	NP
	241*	+	**+**	NP
	261*	+	**+**	NP

Animal with no lesions on the feet were protected against podal generalization (PPG) and animals with lesions on their feet before 7dpc were considered non protected (NP).

* On day 2pc this animal exhibited at least a lesion in snout or mouth. At 7dpc, these animals showed FMDV lesions in the feet, and vesicles in the snout and mouth.

**Positive amplification by RT-PCR of FMDV 3D RNA sequences in tongue epithelium.

A sequence analysis of the capsid coding region corresponding to the B-cell epitope of the FMDV RNA directly recovered from the lesions were performed. A synonymous mutation was found in animal 257 (B-cell epitope unchanged) and a nonsynonymous mutation in animal 264. In the latter, a valine was replace by an alanine at the 144 amino acid position, within B-cell epitope (-1 RGD position)

## Discussion

Synthetic peptides corresponding to protective B- and T-cell epitopes are considered good candidates for safer and more effective FMD vaccines [29]. The group led by Dr. Mowat reported in 1986 for the first and only time the successful protection against FMDV in cattle [6]. The linear peptide spanning the VP1 regions (residues 141 to 158 and 200 to 213 of the serotype O1, 40 residue peptide) synthesized collinearly, induces a neutralizing antibody response and protected two out of three animals vaccinated with a single dose of peptide and three all animals with a revaccination with low dose at 32 days post-vaccination. Nevertheless, all subsequent studies of vaccination with linear peptides on this important FMDV natural host and with a larger number of animals resulted in partial [7] or null protection [30]. Multiple presentation of the B-cell epitope has been proven advantageous over simpler arrangements when eliciting humoral and cellular immune responses [8]. Indeed, a dendrimer peptide displaying four copies of the B cell epitope VP1 (136–154) and one copy of the T-cell epitope 3A(21–40) from type C FMDV, assembled using a thioether conjugation chemistry protects against homologous FMDV challenge in swine [3]. Recently, Blanco et al (2016) reported full protection in pigs vaccinated with the analogous dendrimer B_2_T with sequences from type O FMDV O-UKG assembled using maleimide linkage, an advantageous conjugation chemistry in terms of production simplicity, with positive impact in costs and adaptability [4]. Another important point to highlight is that synthetic peptide with low immunogenicity can promote selection of viral escape mutants as they frequently include a single or a very limited number of B cell neutralizing sites [7]. However, the use of dendrimeric peptides can overcome this potential limitation as these structures allow the incorporation of different B cell epitopes into a single molecule.

Hence, the present report aimed to assess the effectiveness of novel maleimide-conjugated dendrimeric peptides in cattle in affording protective immune responses to FMDV. These new constructs displayed either two or four copies of the B-cell epitope (135–160 from VP1, type O FMDV O1 Campos) and one T-cell epitope 21–40 from VP1 (named B_2_T and B_4_T, respectively). Our results indicate that these two dendrimers afforded 100% PPG to viral challenge.

When compared with the immune response evoked by analogous dendrimers in swine, the main difference observed in bovine is the need for a third immunization to detect consistent levels of neutralizing antibodies. It is possible that the immunogenicity in cattle can be increased by using other adjuvants or different amounts of peptide in the vaccine. Moreover, after a third dose of vaccine containing a low amount of peptide, we observed an enhancement of the IgG affinity rather than an increase on the level of IgGs. The third immunization could have resulted into a more stringent selection of higher affinity B cells, and the emergence of effective neutralizing antibodies.

In addition, pigs vaccinated with dendrimer B_(OUK)2_T_3A_ elicited increased levels of virus-specific IgG1 and IgG2 relative to those of animals vaccinated with B_(OUK)4_T_3A_, while in this study immunization of cattle with B_2_T or B_4_T elicited similar levels of total anti-peptide and anti-FMDV antibodies, as well as high levels of specific IgG1 against the virus. Further experiments are required to understand the low levels of IgG2 detected in this study; noteworthy, the same nomenclature for subclasses among different species often leads to the mistaken believe that these subclasses are homologous and have the same functions [31].

Induction of high levels of FMDV-specific IgG1 subtype in serum has been related to protection in conventionally vaccinated cattle, even at low levels of total IgG [32]. Furthermore, differential IgG1/IgG2 ratios have been associated with protection in animals with low VNT titers [33], as well as in bovines immunized with linear synthetic peptides [7]. Bovine IgG1 is involved in neutralization and also in pathogen opsonization.

Macrophages have a high phagocytic capacity and they can engulf and destroy FMDV, especially if it is opsonized by specific antibodies [27, 34]. At day 44dpv, high opsonophagocytosis levels were found in sera from animals vaccinated with the dendrimeric constructs, with values similar to those from bovines vaccinated with a commercial vaccine.

Animals of the B_4_T group were not only protected from podal generalization but also did not develop lesions in tongue or mouth, near the viral instillation point, which could be related with the trend towards higher opsonization and VNT values observed in sera of these animals when compared with those immunized with B_2_T. One of the two B_2_T-vaccinated cattle that developed tongue lesions (animal 264) showed an amino acid substitution in the region corresponding to the B epitope, which could reflect the selection of a scape mutant in this partially protected animal.

In animals immunized with B_4_T, VNT titers were similar to those of bovines immunized with the commercial vaccine (approved by SENASA for vaccination in Argentina) and associated with Expected Percentage of Protection (EPP) of 80 percent. The EPP estimates the likelihood that cattle would be protected against a challenge of 10.000 bovine infective doses after vaccination [15]. Bovine 253 that elicited low neutralizing antibodies levels (1.2) resulted protected upon challenge, which could be associated with the presence of high IgG1 levels in its serum, in accordance with the results published by Capozzo et al [32].

Importantly, all antibodies induced by the dendrimeric peptides used in this study allowed differentiation of infected from vaccinated animals, since they did not react against the NS protein 3ABC (data not shown), which is the standard diagnostic tool for FMDV replication [35].

It is plausible that the improved immunogenicity of constructs with four versus two epitopes is based on positing repetitive antigens that induce direct cross-linking of surface Ig receptors in immature B cells [36] or that epitope multimerization promotes antigen internalization by dendritic cells or other antigen presenting cells [37]. In any case, B_4_T and B_2_T efficiency in cattle differs from that described for their counterparts in pigs [4] and further experiments are needed to understand these differences among dendrimers that display different B and T cell sequences and target different species. The T-cell epitope VP1 (21–40), previously identified in bovine [11] was incorporated to B_2_T and B_4_T due to the lack of experimental evidence supporting recognition as a T-helper epitope in cattle of the 3A (21–35) epitope included in the dendrimers that successfully protected swine [4].

FMDV usually gains entry through the respiratory tract of cattle [38]. There are a number of mechanisms that have been proposed to explain the production of secretory antibodies after immunization with antigen. One of the mechanisms proposed is the direct diffusion of soluble or phagocytosed antigens to mucosal-associated lymphatic tissues (MALT); other is the migration of activated antigen-presenting cells to MALT from the lymph node that drains the inoculation site.[39, 40]. However, in mucosa, regulation of IgA and IgG1 production in cattle is not completely understood and further studies should be aimed at establishing the association between these immunoglobulins and protection. In animals belonging to B_2_T and B_4_T groups, IgG1 specific against FMDV was increased in nasal secretions, suggesting some role of this immunoglobulin in protection; however, only some animals showed an increased IgA in nasal secretions. It is tentative to speculate the possible implications of both IgG1 and IgA immunoglobulins against viral entry in mucosal tissues.

Animal to animal variation was observed in both the lymphoproliferation and IFN-γ secretion assays, as reported in previous studies with peptide-immunized cattle [7]. Interestingly, B_2_T and B_4_T peptides evoked similar consistent T cell responses, being recognized *in vitro* by lymphocytes from most of the immunized cattle in the proliferation assay, and from all animals in the IFN-γ production assay. The T-cell epitope VP1(21–40] alone was recognized by a lower number of peptide vaccinated bovines, and it was not as efficient inducing cellular responses as B_2_T and B_4_T peptides, which could be due to a better presentation/processing of the dendrimers in the *in vitro* assays used. On the other hand, it has been described that the 135–160 region of VP1 there is recognized as a T-cell epitope in bovine (135–144) [41].

The number of bovines included in this pilot study was similar to those in other introductory studies on vaccine candidates [4, 6, 42, 43], although we were aware that these numbers are not enough for statistical demonstration [19].

In conclusion, our results support that immunization of cattle with dendrimeric peptides such as B_2_T or B_4_T can evoke humoral immune responses similar to those induced by commercial vaccines. More importantly, they were able to elicit protective responses preventing clinical disease. These results supported our new dendrimeric peptide constructs as a promising candidate for peptide subunit DIVA vaccines against FMDV in cattle.
